# Integrating temporal convolutional networks with metaheuristic optimization for accurate software defect prediction

**DOI:** 10.1371/journal.pone.0319562

**Published:** 2025-05-12

**Authors:** Ahmed Abdelaziz, Alia Nabil Mahmoud, Vitor Santos, Mario M. Freire

**Affiliations:** 1 Nova Information Management School (NOVA IMS), Universidade Nova de Lisboa, Campus de Campolide, Lisboa, Portugal; 2 Information System Department, Higher Technological Institute, HTI, Cairo, Egypt; 3 Department of Computer Science, University of Beira Interior Rua Marquês de Ávila e Bolama, Covilhã, Portugal; University of Queensland - Saint Lucia Campus: The University of Queensland, AUSTRALIA

## Abstract

The increasing importance of deep learning in software development has greatly improved software quality by enabling the efficient identification of defects, a persistent challenge throughout the software development lifecycle. This study seeks to determine the most effective model for detecting defects in software projects. It introduces an intelligent approach that combines Temporal Convolutional Networks (TCN) with Antlion Optimization (ALO). TCN is employed for defect detection, while ALO optimizes the network’s weights. Two models are proposed to address the research problem: (a) a basic TCN without parameter optimization and (b) a hybrid model integrating TCN with ALO. The findings demonstrate that the hybrid model significantly outperforms the basic TCN in multiple performance metrics, including area under the curve, sensitivity, specificity, accuracy, and error rate. Moreover, the hybrid model surpasses state-of-the-art methods, such as Convolutional Neural Networks, Gated Recurrent Units, and Bidirectional Long Short-Term Memory, with accuracy improvements of 21.8%, 19.6%, and 31.3%, respectively. Additionally, the proposed model achieves a 13.6% higher area under the curve across all datasets compared to the Deep Forest method. These results confirm the effectiveness of the proposed hybrid model in accurately detecting defects across diverse software projects.

## Introduction

In the initial stages of the software development life cycle (SDLC), defect prediction serves as a fundamental process for ensuring superior software quality [1,2]. This approach is particularly crucial in the context of increasingly intricate and rapidly evolving software systems. The core objective of defect prediction is to pinpoint areas within the software that are prone to defects during the development phase. By identifying these high-risk components, development, testing, and project management teams can effectively prioritize their efforts, thereby improving the software’s overall quality [3,4]. Detecting flawed modules early in the process not only facilitates optimized allocation of resources but also ensures the timely production of reliable software solutions [4]. Additionally, addressing defects in the early phases is vital, as the cost of resolving issues tends to escalate significantly as the project progresses through later stages. Consequently, early defect prediction enhances software reliability and quality while concurrently reducing development expenses [5].

Over the past few decades, extensive research has been conducted on various models for predicting software defects [4,6]. The ability to accurately identify defects depends on the use of diverse metrics, with historical software metrics being particularly crucial in differentiating between defective and non-defective components [7–10]. Studies indicate that selecting specific subsets of these metrics can significantly improve the accuracy and effectiveness of predictive models [11]. Defection plays a critical role in quality assurance, aiding in the production of reliable software while ensuring efficient use of resources and adherence to project timelines. Machine learning methodologies have emerged as powerful tools for early detection defection, as they uncover latent patterns within historical datasets, enhancing predictive accuracy [1].

Software companies continuously seek to develop models capable of accurately assessing the factors contributing to software defects, facilitating improved detection and prediction in software development projects. This research focuses on constructing a robust model to identify critical defect metrics that significantly impact software projects, a topic that remains insufficiently addressed in the current body of literature [12].

Maintaining software quality requires the implementation of various quality assurance practices, including verification, validation, testing, and identifying fault tolerance, with particular focus on software fault prediction. This predictive process estimates potential defects within a software product during and post-development by utilizing predefined metrics or historical defect data from analogous projects [13]. Early detection of software faults, even before project initiation, enables developers to optimize development time and effort. Accurate prediction models are instrumental in identifying defects across different stages of the SDLC, reducing the scope of modules requiring attention at each phase [14].

Defection prediction is a critical aspect of software development, as errors in one version can adversely affect the quality of subsequent versions due to the iterative nature of the development process [14]. The performance of defect prediction models is significantly shaped by the modeling techniques [9] and the selection of metrics employed [15]. Although modeling techniques contribute moderately to performance variations, the impact of metric selection on classification accuracy is comparatively smaller. Developing an effective optimization algorithm requires careful consideration of both the objective function and the solution representation to ensure optimal results [14].

The proposed TCN-ALO model leverages advanced methodologies to effectively address these challenges. TCNs are employed due to their unique features that enhance model performance. Unlike conventional neural networks, TCNs are designed to avoid fully connecting nodes between successive layers, resulting in a substantial reduction in parameters within the convolutional layers compared to fully connected layers. This design makes TCNs particularly well-suited for handling large numerical datasets [16]. By utilizing parameter sharing in the convolutional layers, TCNs process data with fixed-weight filters, further decreasing the number of parameters relative to fully connected layers. Additionally, pooling layers play a crucial role by reducing the dimensionality of the data, which enhances the computational efficiency and adaptability of TCNs [17]. These attributes contribute to the model’s ability to manage complex datasets with improved performance and scalability.

TCNs are highly effective in handling high-dimensional data due to their capacity to learn hierarchical features. The initial layers of a TCN are designed to capture fundamental features, while deeper layers focus on identifying more intricate patterns within the data. One of the key strengths of TCNs lies in their ability to perform automatic feature extraction, eliminating the need for the labor-intensive manual feature engineering process traditionally required in data preprocessing [16].

Researchers have successfully integrated metaheuristic algorithms with predictive models to optimize neural network weights, thereby improving prediction accuracy through hybrid methodologies [18]. These algorithms are often combined with swarm intelligence techniques, including particle swarm optimization (PSO) [19], genetic algorithms (GA) [20], gray wolf optimization (GWO) [21], and bat-inspired algorithms [22]. Such optimization strategies enhance neural networks by effectively mitigating challenges associated with local optima during the adjustment of weights and biases, leading to more robust and accurate predictive models.

ALO is a metaheuristic algorithm inspired by the predatory behavior of antlions, and it has proven effective in diverse optimization tasks, such as feature selection [23] and forecasting [24]. ALO offers several benefits, including minimal reliance on extensive parameter tuning and adaptability to a wide range of optimization problems. However, its exploitation capabilities are somewhat limited, which can result in slower convergence rates [25]. This study adopts ALO as the optimization algorithm due to its well-balanced exploration and exploitation capabilities, which are essential for fine-tuning parameters in convolutional neural networks (CNNs). CNNs are instrumental in software defect detection, leveraging features such as localized connections, parameter sharing, and dimensionality reduction to achieve high predictive accuracy. The integration of ALO enhances CNN performance by optimizing its parameter space, ensuring more effective defect identification in software projects.

While emerging optimizers like Reptile Search, Red Fox Optimization, and Crayfish Optimization demonstrate notable innovation and computational power, ALO remains a preferred choice for tasks requiring high precision and stability over extensive iterations, such as software defect prediction. ALO’s dependable convergence and robust performance make it particularly suitable for these applications. Additionally, unlike many optimizers designed for specific use cases, ALO’s versatility and proven efficacy across a wide range of problem domains establish it as a reliable, general-purpose optimization tool. This adaptability enhances its relevance and effectiveness in various machine-learning applications.

This study utilized the NASA JM1 clean software defect dataset to evaluate performance, employing a 75:25 split for training and testing. This dataset was chosen due to its widespread use in research and its application by software companies for developing predictive models. The primary aim is to develop an optimized model capable of identifying critical defect metrics and addressing software defect challenges effectively. The proposed method demonstrates superior performance compared to state-of-the-art techniques, achieving accuracy improvements of 27.6%, 30.9%, 34.4%, and 29.3% over neural networks, naïve Bayes, decision trees, and random forests, respectively. These findings provide significant insights into resolving software defect issues and enhancing predictive accuracy.

This study presents an integrated model that combines ALO with a TCN to improve software defect prediction. The model iteratively fine-tunes the TCN parameters using ALO, leveraging optimal solutions to achieve enhanced accuracy. The proposed TCN-ALO hybrid model outperforms baseline approaches, including the standard TCN and other machine learning methods, by delivering higher accuracy, sensitivity, specificity, and lower error rates across multiple datasets. Its performance was validated using 22 datasets from the PROMISE repository, demonstrating the model’s robustness and versatility in various defect prediction scenarios. A detailed comparison with advanced methods, such as Gated Recurrent Units (GRU), Bidirectional Long Short-Term Memory (BiLSTM), and Deep Forest, highlights the TCN-ALO model’s superiority in terms of accuracy, efficiency, and predictive capabilities. The results provide valuable insights for deploying the TCN-ALO model in software engineering practices, showcasing its potential to streamline defect prediction processes, reduce development costs, and enhance software quality and reliability.

The structure of this research is organized as follows: Section 2 reviews the existing literature relevant to this field, providing an overview of prior work. Section 3 introduces the proposed model, detailing its design, development process, and distinctive features. In Section 4, the experimental results are presented and thoroughly analyzed to evaluate the model’s performance. Finally, Section 5 concludes the study, summarizing the main findings and discussing their implications for future research and practical applications.

## Related work

Numerous researchers have developed defect prediction models in recent decades using machine learning and statistical techniques. Iqbal et al. [1] evaluated machine learning techniques for early software defect prediction using 12 cleaned NASA datasets. It compares ten classifiers, including Random Forest, support vector machine (SVM), Naïve Bayes, and Decision Trees, assessing their performance through metrics like Precision, Recall, F-Measure, Accuracy, and ROC Area. Results showed that Random Forest and SVM often excelled in accuracy and ROC, though Precision, and Recall exposed issues with class imbalance. The datasets’ imbalance impacted predictions, especially for less frequent defect classes. Accuracy and ROC were found unsuitable as standalone performance measures. The study provides a benchmark for researchers to compare future models. Recommendations include addressing class imbalance, using advanced ensemble methods, and applying feature selection techniques. The findings underline the significance of quality datasets and tailored evaluation metrics in software defect prediction research.

In their 2019 study, Dhanda et al. [5] compared three supervised machine learning techniques: Decision Tree, Naïve Bayes, and Logistic Regression for software bug prediction using historical data. To enhance the predictive capability, they utilized random forest ensemble classifiers and assessed their performance using the K-Fold cross-validation technique. Additionally, Zhou et al. introduced an innovative deep forest model that restructured random forest classifiers into a hierarchical, layer-by-layer framework, leading to notable improvements in defect prediction accuracy across multiple open-source projects. This novel approach achieved a 5% increase in the AUC value compared to conventional machine learning methods, as demonstrated on datasets from NASA, AEM, PROMISE, and Relink.

In their 2019 study, Sutar and et al. [26], proposed a machine learning framework to predict defect-prone areas in software using system testing parameters and a novel “Component Dependency Score (CDS).” Instead of traditional development metrics like lines of code, the study utilizes data such as defect counts, automation percentages, and inter-component dependencies. A Component Dependency Graph (CDG) calculates CDS, reflecting the impact of component dependencies. The model uses decision forest regression, trained and tested in an 80:20 split, achieving 78% accuracy. The approach enabled focused testing on high-risk components, leading to early defect detection and improved resource allocation. Results showed a ±12% deviation in predicted defect counts compared to actual outcomes. The method enhanced test strategies, reduced testing costs, and boosted stakeholder confidence. Plans include creating a regression test recommender and advancing “Shift Left and Shift Down” strategies for better testing efficiency.

In a separate 2019 study, Fan and et al. [27], proposed a deep learning framework for enhancing defect prediction accuracy by analyzing code syntactic and semantic structures. Software Defect Prediction via attention-based Recurrent Neural Network (DP-ARNN) uses Abstract Syntax Trees (ASTs) to capture program context, converting them into tokenized high-dimensional vectors with word embeddings. A Bi-LSTM network learns features from these vectors, while an attention mechanism emphasizes critical patterns for precise predictions. Tested on seven open-source Apache Java projects, the framework outperformed traditional methods like Random Forest and other deep learning models (CNN and standard RNN), with 14% higher F1-measure and 7% higher AUC on average. By leveraging ASTs, it overcomes the limitations of static code metrics, providing developers with actionable insights for defect localization. The study highlights DP-ARNN’s superior ability to distinguish buggy code and suggests further improvements by integrating static metrics and applying the framework to other programming languages.

In a study by Rhmann et al. (2020) [24], explored the use of software change metrics (SCM) and hybrid search-based algorithms (HSBA) for fault prediction in software projects. SCM, such as code churn and line modifications, were extracted from Git repositories of Android versions and used as independent variables. The study compared the performance of HSBAs (GFS-Adaboost and GFS-Logitboost) with machine learning techniques (MLT), including Random Forest, Multilayer Perceptron, and J48. Results showed that GFS-Logitboost outperformed other methods in precision and recall, particularly on larger datasets. A Friedman statistical test indicated no significant difference among techniques for fault prediction. The findings emphasize the potential of HSBAs for accurate fault prediction, but additional experiments on larger datasets and varied languages are recommended to generalize the results. The study highlights the cost-saving benefits of early fault detection in software development. Similarly, Lamba and et al., (2019) [28] a comprehensive comparison was performed employing various machine learning algorithms, including Decision Stump, Linear Regression, Decision Tree, Random Forest, Support Vector Machine, and Neural Network. The evaluation revealed that Support Vector Machines demonstrated superior performance across multiple metrics, such as accuracy, correlation, mean squared error, and R-squared values, surpassing the effectiveness of the other techniques.

In a study by Arora and Saha (2017) [29], evaluated the performance of machine learning techniques, specifically SVM and Artificial Neural Networks (ANN), in predicting software defects. Using seven datasets from the PROMISE repository, the study builds prediction models based on static code and design metrics and assesses them on accuracy, recall, and specificity. Results show that ANN excels in accuracy and specificity, while SVM outperforms in recall, critical for identifying defective modules. The findings emphasize choosing models based on the project’s criticality and evaluation criteria. Similarly, a study by Babu, Himagiri, Vamshi Krishna, Anil Kumar, and Ravi (2019) [30] explored methods to predict software defects, which is essential for improving software quality and reducing costs. It introduces a novel approach combining three cost-sensitive algorithms—Cost-Sensitive Variance Score (CSVS), Laplace Score (CSLS), and Constraint Score (CSCS)—to enhance feature selection. This combined method improves defect prediction accuracy while minimizing misclassification costs compared to applying each algorithm independently. Using datasets from NASA’s Metrics Data Program, the study demonstrates superior performance of the proposed method in precision and sensitivity. The findings suggest that integrating cost-sensitive learning improves defect prediction, aiding in early fault identification and efficient resource allocation.

In a study conducted by Kumudha & Venkatesan in 2016 [31], proposed an adaptive dimensional biogeography-based optimization (ADBBO) model to enhance the performance of Radial Basis Function Neural Networks (RBFNN) in predicting software defects. Using NASA PROMISE datasets, it incorporates cost-sensitive measures to minimize misclassification costs, prioritizing accurate identification of defective modules. The ADBBO-RBFNN model optimizes neural network weights, achieving faster convergence and improved prediction accuracy compared to traditional methods. The results, validated through metrics like sensitivity, specificity, and AUC, demonstrate superior defect prediction capabilities, making it a robust tool for resource-efficient software testing and quality assurance.

Additionally, Yousef (2015) [32] explored the application of data mining techniques to predict software defects by analyzing static software metrics such as size, complexity, and coupling. Utilizing datasets from NASA projects, it evaluates four algorithms—Naïve Bayes, Neural Networks, Association Rules, and Decision Trees—for their predictive accuracy, with Naïve Bayes performing the best individually. A novel weighted voting rule approach combining all algorithms further enhances precision, recall, and overall accuracy. The study also proposes a solution architecture that integrates data mining models with development tools and bug tracking systems to provide real-time feedback to developers on potential defective modules. This integration aims to improve software quality by guiding testing efforts and reducing development costs.

In Malhotra’s 2014 study [33], evaluated statistical and machine learning methods for software fault prediction using static code metrics. It compares logistic regression (LR) with six machine learning (ML) approaches, including Decision Trees (DT), ANN, and SVM, using datasets AR1 and AR6. Results show that ML methods outperform LR in predicting faulty modules, with Decision Trees achieving the highest accuracy (AUC: 0.865 for AR1 and 0.948 for AR6). The study concludes that ML techniques provide better predictive capabilities than traditional methods and suggests their application for enhancing software quality and directing testing resources effectively.

Similarly, in Zheng’s 2010 study [34], evaluated three cost-sensitive algorithms to boost neural networks for software defect prediction using datasets from NASA projects. The algorithms—threshold-moving (CSBNN-TM) and two weight-updating approaches (CSBNN-WU1, CSBNN-WU2)—are evaluated with respect to misclassification costs, measured by Normalized Expected Cost of Misclassification (NECM). The results indicate that CSBNN-TM consistently achieves lower misclassification costs, especially for projects developed in object-oriented languages like C++. It is also more robust to cost ratio estimation errors compared to weight-updating methods. This study concludes that threshold-moving is the most effective approach for building cost-sensitive defect prediction models, providing better adaptability and ease of implementation.

In a study by Singh & Singh Salaria (2013) [35], presented a model leveraging the Levenberg-Marquardt algorithm (LMA) within neural networks to predict software defects during the development lifecycle, focusing on reducing testing costs. Utilizing the PROMISE repository dataset and Chidamber and Kemerer metrics, the study demonstrates an accuracy of 88.09%, outperforming polynomial function-based neural networks (80.3%-78.8%). The developed MATLAB-based GUI includes a feedforward neural network with three hidden layers. The proposed model’s key strength is its high prediction accuracy for fault-prone modules, making it a cost-effective solution compared to traditional techniques. The authors suggest future work to explore alternative training algorithms for further accuracy improvement.

Furthermore, Qiao et al. (2020) [9] proposed a deep neural network model for predicting the number of software defects at the module level, leveraging preprocessing techniques like log transformation and normalization to optimize input data. Evaluated on MIS and KC2 datasets, the approach outperformed state-of-the-art methods such as Support Vector Regression (SVR), Fuzzy SVR, and Decision Tree Regression (DTR), achieving up to a 13% reduction in MSE and a 27% improvement in the squared correlation coefficient (R²). The results demonstrate the model’s capability to capture complex data patterns, significantly improving prediction accuracy and aiding efficient resource allocation during software testing. Training time averaged three minutes, and predictions were made in just seconds, highlighting the method’s practicality and efficiency.

In another work, Kassaymeh, Abdullah, Al-Betar, & Alweshah (2021) [36] introduced a hybrid optimization model combining the Salp Swarm Algorithm (SSA) with a Backpropagation Neural Network (BPNN) to enhance software fault prediction (SFP). This novel SSA-BPNN approach optimizes network parameters to improve prediction accuracy, outperforming traditional BPNN and other state-of-the-art methods on 22 diverse datasets. Key performance metrics like accuracy, sensitivity, specificity, and AUC confirm the hybrid model’s superiority, achieving significant gains, particularly in AUC values, across most datasets. The results demonstrate that SSA’s efficient parameter tuning significantly mitigates BPNN’s limitations, such as slow convergence and local optima, establishing SSA-BPNN as a robust tool for SFP challenges.

Lastly, Sun, Li, Sun, & He (2021) [37] proposed a novel Collaborative Filtering based source project selection (CFPS) method for cross-project defect prediction (CPDP) to address the challenge of selecting appropriate training data for better defect prediction. CFPS uses three steps: (1) similarity mining to compute relationships between a target project and historical projects; (2) applicability mining to assess the relevance of historical projects to each other; and (3) a collaborative filtering algorithm to recommend optimal source projects for training. Experiments with 14 software projects and five classifiers, evaluated using AUC and F-Measure, demonstrate that CFPS consistently outperforms existing methods like EucPS in recommendation performance (e.g., F-Measure), showing improvements of up to 104% in MAP. These results highlight CFPS’s ability to enhance CPDP by leveraging similarities and cross-applicability among projects effectively.

In a recent study, Jin (2021) [38] introduced a Cross-Project Defect Prediction (CPDP) model integrating Domain Adaptation (DA) with kernel twin support vector machines (KTSVM) and optimized through an Improved Quantum Particle Swarm Optimization (IQPSO) algorithm. This approach aims to reduce distribution mismatches between source and target projects for effective defect prediction. Experimental evaluations on 17 open-source software projects demonstrate that the proposed model, DA-KTSVMO, achieves superior performance, often surpassing within-project defect prediction (WPDP) models in F1 scores and AUC metrics when training data is sufficient. DA-KTSVMO consistently outperforms competing CPDP methods, with improvements up to 21.88% in average F1 and significant enhancements in other metrics, validating its effectiveness in leveraging data from different software domains. ML methods have shown considerable promise in improving the processes of predicting and identifying software defects. Current studies focus on enhancing the precision and transparency of these models, as well as ensuring their smooth incorporation into software development workflows, ultimately aiming to minimize defects and elevate software quality.

Although significant strides have been made in applying ML methods to software defect prediction and detection, numerous challenges remain unresolved in existing research. Overcoming these challenges could greatly enhance the efficiency and applicability of ML models in practical software development settings. Achieving this requires a multidisciplinary strategy that combines progress in machine learning, software engineering, human-computer interaction, and economic analysis.

This research presents an innovative methodology that departs from existing advanced techniques, highlighting the efficiency of ALO in optimizing TCN weights. By facilitating both global and local search processes within a single optimization cycle, the TCN-ALO model effectively avoids common issues such as entrapment in local minima, which frequently limit the performance of defect prediction models. This approach is specifically tailored to manage the intricate, high-dimensional feature spaces characteristic of software defect datasets. By consistently outperforming conventional CNN models and other hybrid techniques, the TCN-ALO model demonstrates exceptional versatility in addressing various software defect prediction challenges, marking a significant step forward in software quality assurance practices.

A significant feature of this study is the model’s capacity to achieve optimization with minimal re-training cycles, owing to the efficient convergence characteristics of ALO. This makes the TCN-ALO model both highly accurate and resource-efficient, which is a critical consideration for practical application in real-world software development contexts. Moreover, this paper stands out by offering a comprehensive comparative evaluation, supported by statistical validation methods such as T-tests, against top-performing defect prediction models. The findings demonstrate that TCN-ALO achieves an optimal balance across performance metrics on diverse datasets, highlighting its significance and originality in advancing software defect prediction.

## Materials and methods

**Fig 1:**
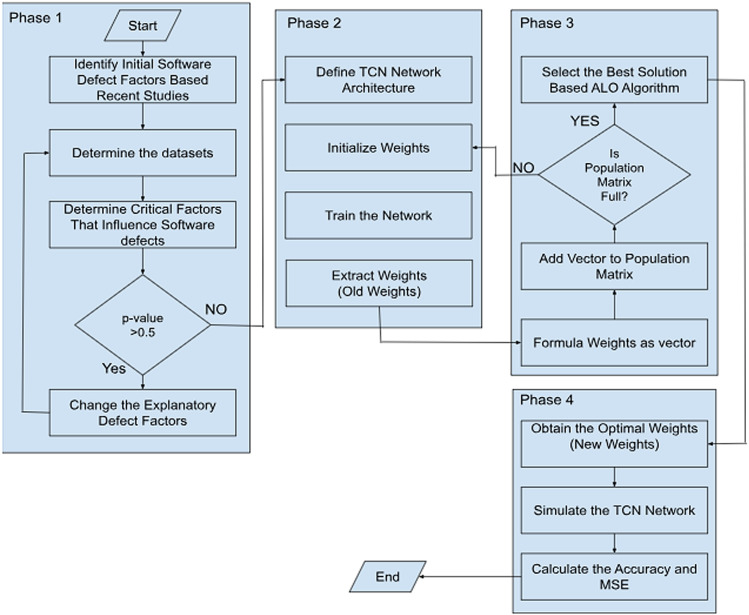
Advanced Predictive Model for Identifying Defects in Software Projects.

This section introduces the TCN-ALO model, a novel framework for predicting defects in software projects. The model comprises four distinct phases, as depicted in Fig 1:

- Conduct a review of recent studies to identify key factors associated with software defects and apply data preprocessing techniques.
- Leverage ALO to optimize the weights of TCN, improving both accuracy and mean squared error (MSE).
- Implement the ALO-TCN model to predict defects in software projects.

Each phase of this advanced methodology is detailed comprehensively in the subsequent sections.

### Dataset

Software defects can lead to operational failures, and addressing these issues during the later stages of development is often expensive and time intensive. Identifying defects early in the development process improves software reliability, quality, and efficiency while simultaneously lowering production costs [5]. An effective approach for defect detection involves utilizing various metrics to classify software modules as either defective or non-defective [7–10]. Research has consistently shown that selecting specific subsets of software metrics can significantly enhance the accuracy and performance of classification models [11]. Table 1 provides details about multiple datasets used for evaluating software defect prediction models. It includes the following columns for each dataset:

**Table 1:** Description of Datasets.

Dataset	Feature No.	Defects No.	Defects (%)	Dataset	Feature No.	Defects No.	Defects (%)
**KC1**	22	325	15.50	**TomGat-6.0**	20	77	8.97
**KC2**	21	107	20.49	**MW1**	37	31	7.69
**KC3**	39	43	9.38	**JEdit-4.0**	20	75	24.50
**JM1**	22	1759	18.34	**JEdit-4.2**	20	48	13.08
**AR1**	29	8	7.40	**JEdit-4.3**	29	10	2.03
**AR3**	29	8	12.70	**Ant-1.7**	20	166	22.28
**AR4**	29	20	18.69	**PC1**	37	76	6.94
**AR5**	29	8	22.22	**PC2**	36	23	2.15
**AR6**	29	15	14.85	**PC3**	38	140	12.40
**CM1**	22	49	9.83	**PC4**	38	178	12.72
**MC2**	39	52	32.29	**PC5**	38	516	3.00

- Dataset Name: Identifies the dataset, often corresponding to specific software projects or versions.
- Feature No.: Indicates the number of features (attributes or metrics) included in the dataset for analysis.
- Defects No.: Shows the total number of defective instances (e.g., faulty modules or components) within the dataset.
- Defects (%): Represents the percentage of defective instances relative to the total number of instances in the dataset.

KC1, KC2, KC3: These datasets, part of NASA’s software projects, vary in feature counts and defect percentages, with KC2 having the highest defect rate at 20.49%. JM1: This dataset includes a substantial number of defective instances (1759) and a defect rate of 18.34%. AR1 to AR6: These datasets belong to the AR project and exhibit differences in feature numbers and defect rates, with AR6 showing a relatively high defect rate of 14.85%. CM1, MC1, MC2: Another set of NASA datasets featuring varying defect rates, with MC2 having the highest defect percentage at 32.29%. TomCat-6.0: A smaller dataset with 20 features and 77 defective instances, resulting in an 8.97% defect rate. MW1: A compact dataset with 37 features and a 7.69% defect rate. JEdit (4.0 to 4.3): Successive versions of the JEdit project show progressively lower defect percentages. Ant-1.7: This dataset includes 166 defective instances, equating to a defect rate of 22.28%. PC1 to PC5: Versions of the PC software project with defect percentages ranging from 2.15% (PC2) to 12.72% (PC4).

This research leverages software metrics as crucial instruments for identifying defective and non-defective modules in software projects. These metrics, extracted from different aspects of the software’s code base, architecture, and past performance, play a vital role in predicting defects. Commonly used software metrics encompass examples such as:

- Lines of Code (LOC): A fundamental metric that counts the total number of lines in the software. Larger codebases are often associated with a higher likelihood of defects.
- Lack of Cohesion of Methods (LCOM): Evaluates the degree of disconnection among a class’s methods. A high LCOM value suggests low cohesion, indicating a higher potential for defects.
- Weighted Methods per Class (WMC): Represents the total complexity of all methods within a class, serving as an indicator of defect density.
- Depth of Inheritance Tree (DIT): Captures the depth of a class within an inheritance hierarchy. Greater depth can increase complexity, making defects more likely.
- Number of Changes (NOC): Tracks how frequently a module or file has been modified. Modules with higher change frequencies are typically more prone to defects.

### Analysis of research and implementation

**Fig 2:**
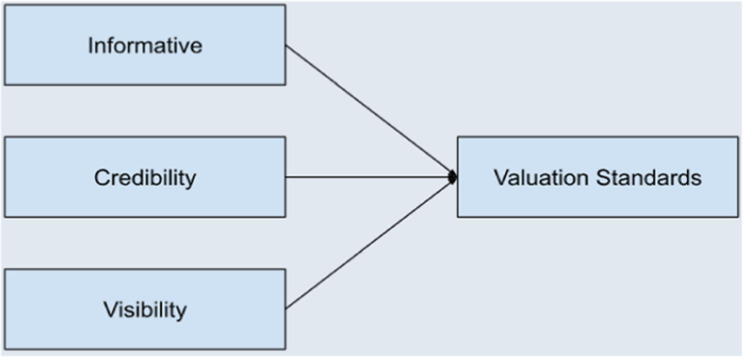
Assessment Standards for Determining the Preliminary Set of Defect Factors.

The first step entails analyzing recent research to identify defects in software projects and creating a preliminary list of factors associated with these defects. This list is developed based on defined evaluation criteria, including informativeness, credibility, and visibility, as depicted in Fig 2 [39].

In addition, Fig 2 illustrates the evaluation criteria used to identify an initial list of defect factors where the goal is to establish valuation standards that help in assessing or identifying defect factors effectively. These standards serve as a benchmark or guideline for evaluation. The process involves three evaluation criteria that influence or feed into the valuation standards:

- Informative: this criterion ensures that the data or information about defect factors is detailed, descriptive, and provides sufficient context. It helps in making the evaluation process robust and meaningful.
- Credibility: this criterion ensures that the sources or evidence supporting the defect factors are reliable, accurate, and trustworthy. This factor is critical in avoiding misleading or incorrect evaluations.
- Visibility: refers to the ease with which defect factors can be observed, measured, or recognized. It emphasizes the importance of identifying factors that are noticeable and accessible for analysis.

Data preprocessing is the process of transforming raw data into a structured format that is ready for analysis and machine learning applications. This often requires the application of specific techniques or formulas. Listed below are key preprocessing steps, along with their corresponding formulas and descriptions:

#### Data cleaning.

Data cleaning is the process of eliminating duplicate entries, errors, anomalies, missing values, and inconsistencies in a dataset. This procedure enhances the dataset’s caliber and dependability for further analysis.

#### Feature scaling.

Feature scaling is a data preprocessing technique used to standardize the range of independent variables or features in a dataset. It ensures that all features contribute equally to a model and prevents those with larger numerical values from dominating the results. Feature scaling is particularly important for algorithms that compute distances [20]. The formula for Min-Max Scaling is given as Eq (1):


$$\text{Min-Max scaling: }\left(\text{x }-\text{min}\right)\text{/ }\left(\text{max-min}\right)$$ (1)

- x: The original value of the feature.
- min: The minimum value in the feature’s range.
- max: The maximum value in the feature’s range.
- Min-Max scaling: The scaled value after transformation

#### Distribution dataset.

The distribution represents the frequency or probability of different outcomes in the dataset. It shows how often certain values or ranges of values occur for a given variable [40]. There are many types of Distributions, as follows:

- Uniform Distribution: All values are equally likely to occur. For example, rolling a fair six-sided die.
- Normal Distribution (Gaussian): A symmetric bell-shaped curve where most data points cluster around the mean. Many natural phenomena follow this distribution (e.g., heights, test scores).
- Skewed Distribution: A distribution that is not symmetric. It can be:
- Left-skewed (negative skew): Tail extends to the left.
- Right-skewed (positive skew): Tail extends to the right.
- Bimodal Distribution: Two distinct peaks or modes in the data.
- Multimodal Distribution: More than two peaks.

It is very important due to feature scaling, selected models, and data transformations. Understanding the distribution of a dataset is crucial for designing effective models, applying the right preprocessing techniques, and making accurate interpretations of the results. By analyzing the distribution, you can better understand the structure of the data, handle potential issues like outliers or skewness, and choose the most suitable algorithms for your problem.

### Antlion optimization

Antlions exhibit an intricate hunting strategy that serves as the inspiration for the ALO, a unique meta-heuristic algorithm grounded in swarm intelligence. Their hunting mechanism is characterized by five distinct phases: executing a random walk, constructing a trap, capturing an ant, sliding the prey toward the antlion, securing the prey, and reconstructing the trap. For an in-depth discussion of this approach [41].

In the initial stage, ants utilize a random walk algorithm to navigate the parameter space, with antlions serving as key agents in steering them toward potentially optimal regions. The initial populations for both ants and antlions are randomly initialized, as outlined in Equation (2) [41].


$$\text{x = rand × }\left(\text{UPB}-\text{LOB}\right)\text{+ LOB,}$$ (2)

This equation defines the lower and upper bounds of the problem’s parameters as LOB and UPB respectively. The position of each ant and antlion within the D- dimensional search space is represented by a vector of size D. To emulate trap construction, the algorithm employs the roulette wheel selection (RWS) mechanism to choose a target antlion. Antlions with superior fitness are assigned higher probabilities of selection, thereby enhancing their likelihood of capturing ants. The random walk process for each ant during every iteration of the algorithm is depicted in Equation (3):


$$\text{X}\left(\text{t}\right)\text{= }\left[\text{0, Csum}\left(\text{2r}\left({\text{t}}_{\text{1}}\right)-\text{1}\right)\text{,}...\text{, Csum}\left(\text{2r}\left({\text{t}}_{\text{T}}\right)-\text{1}\right)\right]\text{,}$$ (3)

In this context, r(t) is assigned a value of 0 when a randomly generated number is less than or equal to 0.5, and 1 otherwise. Csum denotes the cumulative sum, while T represents the total number of iterations. To ensure that the ant remains within the boundaries of the search space, the process is normalized using the defined upper and lower limits, as outlined in Equation (4):


$$X_{i}^{t}=\frac{\left(X_{i}^{t}-a_{i}\right)*\left(b_{i}-c_{i}^{t}\right)}{\left(d_{i}^{t}-a_{i}\right)}+c_{i}$$ (4)

In this equation, $a_{i}$ and $b_{i}$ represent the minimum and maximum values of the random walk for the i-th variable while $c_{i}^{t}$ and $d_{i}^{t}$ indicates the minimum and maximum values of the i-th variable at the t-th iteration. To capture the effect of the antlions on the ants’ movement, we employ Equation (5):


$$\begin{array}{l} c_{i}=\text{antlion}+c_{i}\\ d_{i}=\text{antlion }+d_{i} \end{array}$$ (5)

The movement of each ant is influenced by the presence of two antlions. The first antlion, chosen through the RWS, is regarded as the winner, while the second, based on fitness assessment, is the top-performing antlion. The dynamic changes in the locations of the ants during each cycle are captured in Equation (6):


$$\;Ant_{i}^{t}=\;\frac{R_{A}^{t}+R_{E}^{t}}{2}$$ (6)

In this equation, $Ant_{i}^{t}$ denotes the position of the i-th and the t-th iteration. The term $R_{A}^{t}$ represents the random walk around a specific antlion during the t-th iteration, while $R_{E}^{t}$ refers to the random walk around the elite antlion from that iteration. To facilitate the guiding process of the ants towards the trap—also referred to as converging to an antlion—the algorithm gradually reduces the limits of the following variables throughout the iterations, as detailed in Equation (7):


$$c^{t}=c^{t}/\text{I}$$

$$d^{t}=d^{t}/\text{I}$$ (7)

In this context, the shrinkage factor I is set to one for the initial ten percent of all repetitions. Following this phase, I is calculated using the formula I = 10^k^ $\frac{t}{T}$, as illustrated in Table 2. The specific value of the constant k is determined based on the current iteration, as outlined in the same table. This adaptive approach enhances the algorithm’s efficiency and effectiveness in locating optimal solutions.

**Table 2:** The value of constant k varies based on the iteration being executed.

Current Iteration t	Constant k
Greater 10 percent (T)	2
Greater 50 percent (T)	3
Greater 75 percent (T)	4
Greater 90 percent (T)	5
Greater 95 percent (T)	6

At the end of each iteration, the process concludes with the prey being captured and the trap being reconstructed as the final step. If an ant demonstrates superior performance compared to an antlion, the roles are reversed, and the antlion adopts the position of the ant.

In algorithm 1 randomly generates the positions for *n ants and n antlions as the starting points within the search space*. Calculate the fitness values of all ants and antlions based on the fitness function (Loss function). Identify the antlion with the highest fitness value, as it influences the ants’ movement. The main loop runs until the maximum number of iterations (MaxIter) is reached. For Each Ant (Sub-loop): Select an antlion using the RWS method and this simulates the antlion building a trap, Assist the selected antlion in attracting ants (simulate prey behavior). The process is guided by Equation (5), which Simulates the random walk of the ant and normalizes its position within the bounds of the search space using Equations (6) and (7). After completing the random walk and movement, recalculate the fitness values of all ants. Replace any antlion that is outperformed by an ant with the superior ant, simulating the role reversal between prey and predator. Update the elite (the best antlion found so far) if a new antlion surpasses its performance. Repeat steps 5–7 for each ant and proceed to the next iteration. After completing all iterations, determine the position of the best antlion, which represents the optimal weights in the TCN. Algorithm 1 outlines the formulation of ALO.

**Algorithm 1. An Algorithm of ALO to Identify the Optimal Weights in the TCN**

**Input: Input: Search Space,**

**Fitness Function,**

**Ants and ant lions,**

**Iterations (Max_Iter_), a_i_,b_i_**

**Output: The best ant lion and its fintness**_**(Optimal Weights in TCN)**_

1. Set a random number of *n* ant positions and *n* ant lion positions as the starting point.

2. Calculate the fitness of all ants and ant lions.

3. Determine the best ant lion

4. **While t <= Max**_**Iter**_ **do**

5.  **For all** *ant*_i_ **do**

6.   Determine the ant lion (building trap) by using RWS; refer to Eq (3,4)

7.   Assist the ant lion in attracting the ants; refer to Eq (5)

8.   Establish a random walk for anti and normalize it; refer to Eq (6,7)

9.  **End For**

10.   Calculate the fitness of all ants.

11.   More robust ant should be used instead of an ant lion.

12.   Update the elite if an ant lion becomes fitter than the elite.

13. **End While**

14. **Determine which posture of the ant lion is the best**_**(Optimal Weights in TCN)**_

The hybrid TCN-ALO model demonstrates substantial performance improvements, particularly in addressing complex challenges such as software defect detection. By combining TCN-ALO, the model achieves superior parameter tuning, resulting in notable enhancements in critical metrics like accuracy, sensitivity, and specificity. These improvements are not marginal; the hybrid model consistently surpasses the performance of traditional TCNs and other sophisticated approaches, including recurrent neural networks and various deep learning techniques. Its precision, observed across diverse datasets, highlights the model’s robustness and adaptability. Consequently, the TCN-ALO model effectively reduces error rates and enhances detection capabilities, establishing itself as a powerful and reliable solution for defect prediction and other high-stakes applications requiring precision and dependability.

#### ALO population construction.

Step 1: Representing weights and biases as a vector involves constructing a vectorized format to encapsulate the updated weights and biases derived from the initial implementation of the TCN in the previous phase. This vector serves as an individual solution for the ALO in the next stage of the model. The vector is expressed as X = (W1, W2,……, Wzm, b1, b2), where *z* denotes the number of nodes in the input layer, and *m* indicates the number of nodes in the hidden layer.

Step 2: Incorporating vectors into the population matrix is carried out iteratively to establish the initial population for the ALO. The structure of the initial population matrix for the ALO is outlined as follows:

The initial population matrix for the ALO adheres to a structure of *n × d*, consisting of *n* distinct solutions, each having *d* dimensions.


$$\left[\begin{array}{ccccc} x_{1}^{1} & x_{2}^{1} & x_{3}^{1} & \cdots & x_{\text{d}}^{1}\\ x_{1}^{2} & x_{2}^{2} & x_{3}^{2} & \cdots & x_{\text{d}}^{2}\\ \vdots & \vdots & \vdots & \vdots & \vdots \\ x_{1}^{\text{i}} & x_{2}^{\text{i}} & x_{3}^{\text{i}} & \cdots & x_{\text{d}}^{\text{i}}\\ \vdots & \vdots & \vdots & \ddots & \vdots \\ x_{1}^{\text{n}} & x_{2}^{\text{n}} & x_{3}^{\text{n}} & \cdots & x_{\text{d}}^{\text{n}} \end{array}\right]$$

The initial population matrix for the ALO follows a template where the structure of the matrix is (n*d), composed of n solutions with d dimensions, each being unique.

#### ALO improvement.

ALO can enhance the weight optimization process in TCNs by effectively tuning the model’s parameters. ALO leverages the predatory behavior of antlions to guide the search for optimal weights, using an iterative process to explore the parameter space. By incorporating this swarm-based optimization technique, ALO can fine-tune the weights in TCNs more efficiently, improving performance metrics such as accuracy and convergence speed. This results in more precise temporal feature extraction, enhancing the overall effectiveness of the TCN in tasks like time series prediction or sequence modelling.

### Temporal convolution network

A **TCN** is a type of deep neural network specifically designed to model sequential data. It uses causal convolutions to ensure no future information is leaked into the current time step, making it suitable for tasks like time series forecasting, TCN has important key characteristics, as follows [16]:

- **Causal Convolutions:** Ensures that the output at time t is only influenced by inputs from time t and earlier.
- **Dilated Convolutions:** Expands the receptive field exponentially without increasing the number of parameters.
- **Residual Connections:** Helps in training very deep networks by avoiding gradient vanishing.
- **Fully Convolutional:** Only uses convolutions, no recurrent units, for efficient parallel processing.

It has four main components as follows [17]:

- **(a) 1D Convolutions**

1D convolutions are the foundation of TCNs. For a single-layer convolutional network, the output at time t, denoted as Yt, is computed as Eq (8):


$$\text{Yt = }\sum_{K=0}^{K-1}w_{k}.x_{t-k}$$ (8)

Where:

- K is the kernel size.
- are the weights of the kernel.
- $x_{t-k}$is the input at time t−k.

- **(b) Causal Convolutions**

In causal convolutions, the filter ensures that the output at time t only depends on the current and past inputs:


$$\text{Yt}=\;\sum_{K=0}^{K-1}w_{k}.x_{t-k}\text{ for t}\geq \text{k }$$ (9)

This prevents information from future time steps from affecting the current output.

- **(c) Dilated Convolutions**

Dilated convolutions allow the network to have an exponentially larger receptive field without increasing the number of parameters or computational cost. The dilation rate d determines the spacing between filter elements. The equation becomes:


$$\text{Yt }=\sum_{K=0}^{K-1}w_{k}.x_{t-d.k}$$ (10)

For example:

- 1d=1: Standard convolution.
- 2d=2: Skips every second input.
- 4d=4: Skips every fourth input.

This exponentially increases the receptive field as the dilation rate increases.

- **(d) Residual Connections**

Residual connections are used to stabilize training in deep TCNs. The output of a residual block is given by


$$\text{Output = Activation }\left(f\left(x\right)+x\right)$$ (11)

Where *f(x)* is the output of the dilated convolutional layer(s).

Algorithm 2 for using a TCN to predict software project defects begins with defining the architecture of the TCN layers. The input parameters include the sequence length, the number of layers, kernel size, dilation rates, and activation functions. For each layer, a dilated causal convolution is performed to capture temporal dependencies while preserving causality. This ensures that the output at a given time step depends only on the current and past inputs, not future ones. The dilation rate, which increases exponentially across layers (D), enables the network to expand its receptive field efficiently, capturing long-term dependencies in the data. After each convolution, a non-linear activation function, such as ReLU, is applied to introduce non-linearity into the model. Residual connections are then added, combining the input of a layer with its output to prevent the vanishing gradient problem and allow for stable training even in deep networks.

Once the TCN layers have been defined, the algorithm transitions to the final layer, where a 1D convolution is applied to map the output of the residual TCN layers to the desired output dimension. This layer reduces the intermediate outputs into a prediction format that aligns with the task at hand—whether it’s classification, regression, or another predictive objective. By using a kernel size of 1 in this layer, the network simplifies the output while preserving the information learned from the previous layers. This step allows the TCN to output defect predictions based on temporal dependencies encoded in the input features. Finally, the algorithm evaluates the performance of the trained model on a validation or test set. Metrics such as accuracy and loss are computed to gauge the model’s effectiveness in predicting defects. These metrics provide critical feedback for refining the model and tuning hyperparameters during training. After achieving satisfactory performance, the optimized TCN model’s parameters (weights) are saved for reuse, particularly for integration with the Antlion Optimization (ALO) algorithm, which enhances the system’s efficiency in revealing software defects. This step ensures that the trained model can be applied to real-world scenarios, emphasizing its adaptability and practical value for defect prediction in software projects.

**Algorithm 2. An Algorithm of the TCN to Reveal Defects of Software Projects.**

**Input:**

**- Input sequence length (L)**

**- Number of layers (N)**

**- Kernel size (K)**

**- Dilation rates (D = [1, 2, 4, ..., 2^(N-1)])**

**- Number of filters per layer**

**- Activation function (e.g., ReLU)**

**Output: £ (Predicting defects of software projects)**

**1. Define TCN layers:**

**- For i in range(N): # Loop over N layers**

**a. Perform dilated causal convolution:**

**- Output = Convolution1D(X, Kernel_Size=K, Dilation_Rate=D[i])**

**b. Apply activation function:**

**- Output = ReLU(Output)**

**c. Add residual connection:**

**- Residual_Output = Output + X (Ensure dimensions match)**

**d. Update X with Residual_Output.**

**2. Final layer:**

**- Apply 1D convolution to map the output to the desired dimension:**

**- Output = Convolution1D(Residual_Output, Kernel_Size=1)**

**3. Model Evaluation:**

**- Evaluate the trained model on a validation set or test set:**

**accuracy = Accuracy(predictions, y_true)**

**loss = Loss(predictions, y_true)**

**- Print the current epoch, accuracy, and loss metrics.**

**4. Save the Trained Model:**

**- Store the optimized TCN parameters (weights and biases) for future use in**

**ALO algorithm.**

**Return £**

### TCN simulation

The process of implementing and simulating a TCN with optimized parameters follows several key steps aimed at enhancing fault detection accuracy as follows:

In the initial stage, the ALO is employed to identify the most effective parameters for the TCN. This process involves generating new weights and biases designed to improve the network’s overall performance. The ALO algorithm treats these weights and biases as a collection of vectors, where each vector represents a possible solution in the parameter space. The optimization process iterates through various configurations, refining the parameters to determine the most optimal combination for enhancing the TCN’s capabilities.

Once the optimal weights and biases have been identified, they are integrated into the TCN along with the simulation input data. This dataset consists of sample fault data, specifically designed for both the training and testing phases of the TCN. The input data undergoes a pre-processing phase to ensure its accuracy, consistency, and alignment with the fault detection objectives, thereby making it suitable for the model’s evaluation and learning tasks.

In this stage, the TCN is simulated using the previously determined optimal parameters. The network processes the input data to generate predictions, which are subsequently analyzed to assess the model’s accuracy. The estimated error, denoted as *e*, is calculated based on the discrepancies between the predicted and actual outcomes, indicating the model’s performance during the simulation phase.

The accuracy of the predictions was assessed by calculating the estimated error, denoted as *e*. The Error Rate Ratio (ERR) was primarily used as the key metric for evaluating accuracy, along with other performance indicators discussed in the Results Section. To determine the accuracy of the forecasts, the predicted outcomes from the TCN were compared against the target values derived from the simulation, enabling a thorough evaluation of the model’s performance.

Algorithm 3 demonstrates how **ALO** can be used to optimize the weights of a **TCN, as follows**:

**Algorithm 3. Proposed Model: TCN-ALO**

 **1. Initialize ALO parameters:**

  **- Population size (N) (number of ants and antlions)**

**- Maximum iterations (MaxIter)**

  **- Search space (weight range for TCN, e.g., [-1, 1])**

  **- Randomly initialize the positions of ants (candidate weights for TCN)**

  **- Randomly initialize the positions of antlions (guiding weights for TCN)**

 **2. Evaluate fitness of each ant and antlion:**

  **- For each ant/antlion:**

   **a. Assign the candidate weights to the TCN.**

   **b. Train the TCN for a small number of epochs or a single forward pass.**

   **c. Compute the fitness using the validation loss or accuracy:**

    **- Fitness = -Validation Loss or +Accuracy.**

 **3. Set the best antlion (Elite) as the one with the best fitness.**

 **4. Begin optimization loop (for iteration = 1 to MaxIter):**

   **a. For each ant:**

    **- Perform a random walk influenced by a selected antlion.**

    **- Update the ant's position (weights) within the search space.**

   **b. Evaluate fitness of each updated ant:**

    **- Assign the new weights to TCN.**

    **- Compute the validation loss or accuracy.**

   **c. Update the positions of antlions:**

    **- If an ant has better fitness than its paired antlion, replace the antlion with the ant.**

   **d. Update the Elite:**

    **- If a better solution is found, update the Elite with the best-performing antlion.**

 **5. Output the Elite as the optimized weights for the TCN.**

 **6. Assign the optimized weights (Elite) to TCN.**

 **7. Train the TCN using the optimized weights for full training on the dataset.**

 **8. Return the trained TCN model with the optimized weights.**

In algorithm 3, there are many steps as follows:

- Initialization: Ants and antlions are initialized as random sets of weights for the TCN. The search space defines the range of possible weights (e.g., between -1 and 1). Each candidate weight configuration represents a potential solution.
- Fitness **Evaluation:** The fitness function evaluates the TCN’s performance (e.g., validation accuracy or loss) using the current set of weights. A small number of training iterations or forward passes are used to calculate this score.
- Optimization **Process:**
  - Ants perform random walks influenced by the antlions, searching for better weight configurations.
  - The antlions guide the search, and their positions are updated if an ant finds a better solution.
  - The Elite represents the globally best-performing weight configuration and is updated iteratively.
- Final **Output:** After completing the optimization loop, the best weight configuration (Elite) is assigned to the TCN. The TCN is then trained fully on the dataset to refine these weights further.

## Experimental results

**Fig 3:**
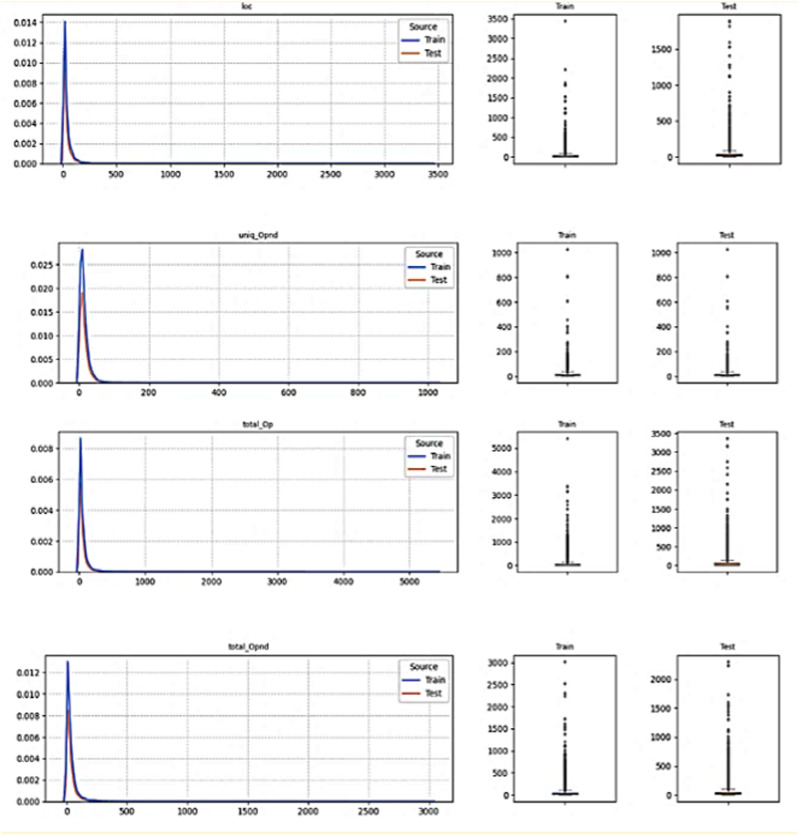
Sample of Distribution Numerical Feature in the List of Defect Factors Before the Feature Scaling.

**Fig 4:**
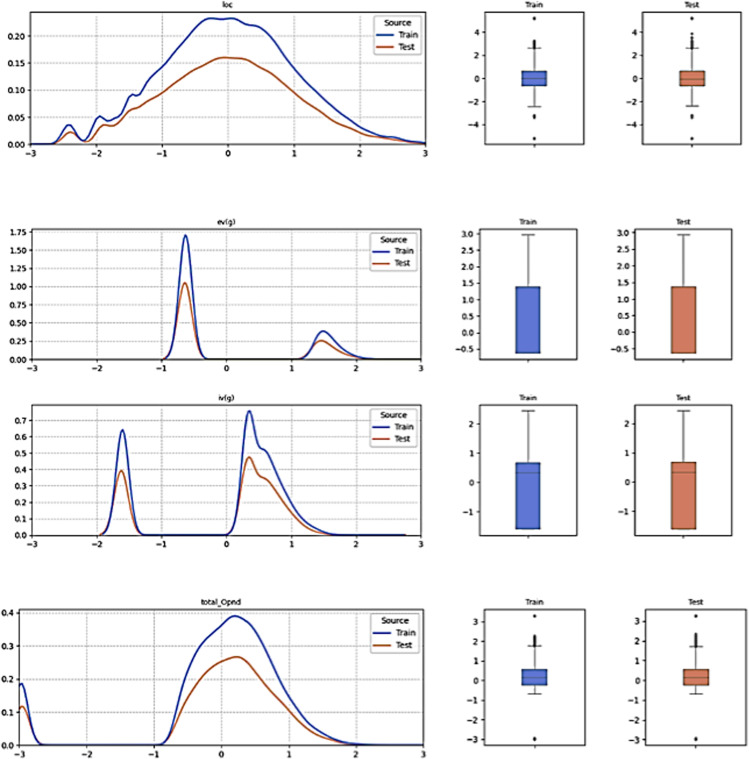
Sample of Distribution of Numerical Feature in the List of Defect Factors After the Feature Scaling.

The following section details the empirical findings of our proposed methodology, which incorporates three distinct techniques: ALO and TCN. The approach initiates with several pre-processing steps. Initially, the attributes of the dataset are categorized, with defect factors in software projects designated as independent variables and the extent of their influence on software defects as the dependent variable. Next, the dataset is partitioned, allocating 70% for training and 30% for testing. The dependent variable is then converted from categorical values (False, True) to binary values (0, 1). Following this, the independent variables are normalized to a range of 0–1. A crucial step in this process is thoroughly understanding the distribution of each numerical feature in the dataset, which allows for the identification of outliers, management of skewness, evaluation of the normality assumption, determination of feature importance, and selection of appropriate statistical techniques as shown in Figs 3 and 4. These steps collectively improve the model’s ability to accurately predict software defects and enhance its overall robustness.

### Results of ALO and TCN

The configuration of the ALO algorithm in this study was carefully designed to ensure optimal performance and consistent results. The setup incorporates the following key parameters:

ALO algorithm in this study utilized a population size of 30 solutions. This configuration was selected to achieve a balance between the exploration of the search space and the exploitation of promising areas. By maintaining sufficient diversity within the population, the setup ensures an effective search process while enabling efficient convergence toward optimal solutions.

The algorithm was executed across 200 iterations, enabling a step-by-step refinement of candidate solutions. This iterative approach facilitated systematic navigation through the search space, ensuring the effective optimization of the targeted parameters and progressively enhancing solution quality.

The range or bounds for the variables to be optimized: [-100, 100]. The method is used to select antlions during each iteration (RWS). The stopping conditions, such as a threshold for improvement or maximum steps (Max Iterations).

The objective function for the ALO algorithm was formulated to minimize the loss function (LFOF) associated with the hybrid model’s efficiency in detecting software defects. The primary goal was to reduce the classification error rate, which was calculated using the following methodology, Eq(12) [42]:


$$\text{LOFO }=\frac{1}{N}\sum_{i=1}^{N}Loss\left(Y_{i},M_{i}\right)$$ (12)

Where N is the total number of instances, Yi represents the actual labels, and Mi denotes the predicted labels by the model. This formulation effectively guided the ALO algorithm’s search for optimal parameter settings that enhance the models’ predictive accuracy.

In this study, we employed the ALO algorithm to fine-tune several key parameters that significantly influence the performance of our hybrid model. The optimization concentrated on three critical areas: the learning rate, batch size, and the number of convolutional layers.

Firstly, we meticulously optimized the learning rate, which is crucial for determining the step size during the optimization process, within a specific range of 0.0001 to 0.1. This carefully selected range enables us to achieve a balance between rapid convergence and stability throughout the training phase.

Next, we evaluated the batch size, which defines the number of training examples processed in a single iteration. We varied this parameter between 16 and 128 to assess how different batch sizes impact training efficiency and the model’s capacity to generalize from the training data.

Lastly, we optimized the number of convolutional layers—a critical factor that dictates the model’s ability to learn intricate features from the data—within the range of 1–5 layers. This range provides the necessary flexibility to evaluate the trade-offs associated with increased model complexity and the related risk of overfitting.

By utilizing the ALO algorithm, we systematically adjusted these parameters to identify the optimal configuration that yielded the highest detection performance for identifying software defects.

**Fig 5:**
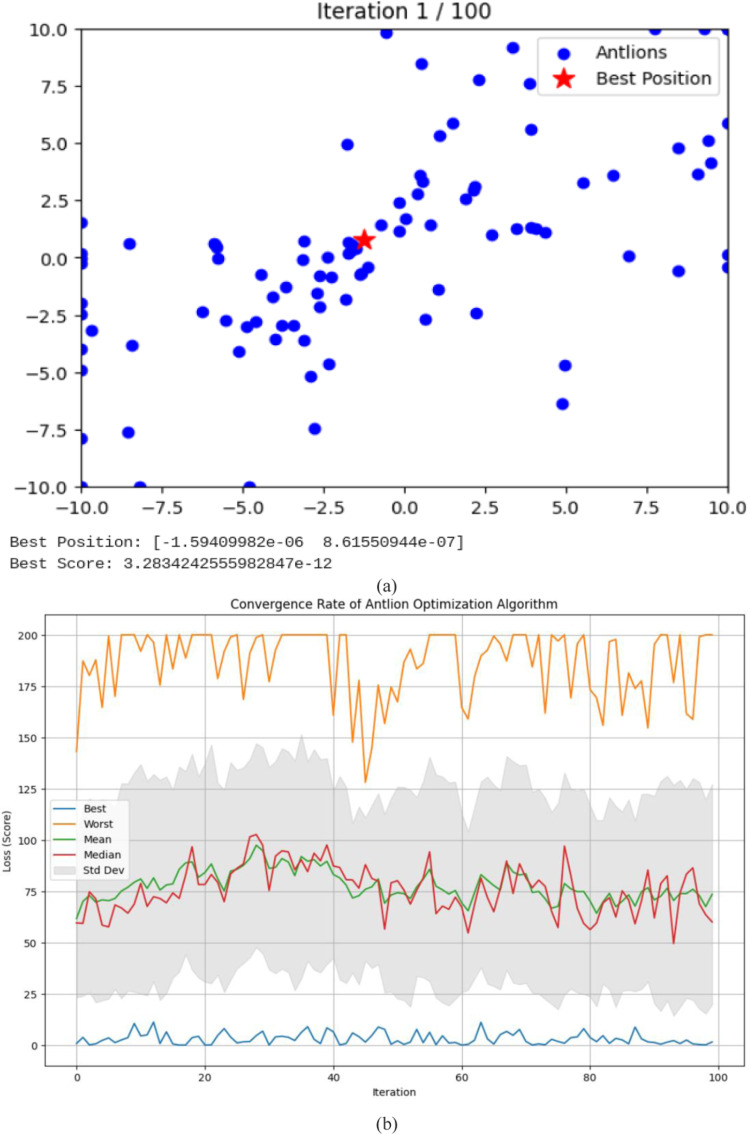
The Results of ALO in terms of (a) - Search history, and (b) - convergence rate.

In Fig 5, the optimization trajectory highlights the antlion’s position throughout the search process, demonstrating that the ALO algorithm effectively investigates potential regions of the search space across all evaluated test functions.

The dynamics of the ALO algorithm when addressing composite test functions showcase distinct patterns, particularly in how it balances exploration and exploitation within the search space. At the outset, the algorithm performs random walks with expansive boundaries, facilitating comprehensive exploration of the search space. Over time, these boundaries gradually shrink, focusing the search on potentially optimal regions. This adaptive process allows the algorithm to transition from broad exploration to targeted exploitation, enhancing its ability to converge on the optimal solution with greater precision.

This adaptive narrowing process plays a crucial role in the optimization mechanism, enabling the ALO algorithm to effectively shift from exploration to exploitation and enhancing its chances of locating the global optimum. A consistent reduction in the average fitness of the antlions across all test functions demonstrates the algorithm’s systematic improvement of the initial random population. This improvement is further reflected in the convergence curves, which progressively approach the global optimum with greater accuracy as iterations proceed. Notably, the later stages of the iterations exhibit an accelerated convergence rate, as the algorithm focuses on localized search, allowing it to efficiently refine and identify optimal solutions.

Fig 5(a) illustrates the initial exploration phase of the ALO algorithm, characterized by a wide distribution of candidate solutions (antlions) and the early detection of a highly promising position (optimal solution). As the optimization process progresses, the antlions are anticipated to gradually converge toward this optimal position, narrowing the search space and enhancing the quality of solutions in subsequent iterations. Fig 5(b) highlights key observations: during the initial phase, the worst solutions display erratic behavior, while both the mean and median values are relatively high, accompanied by a large standard deviation. This stage reflects the exploration phase, where the algorithm evaluates diverse solutions across a wide range of loss values. Between iterations 30 and 70, the mean and median begin to stabilize, and the standard deviation decreases, signifying a shift from exploration to exploitation as the algorithm focuses on refining promising solutions. From iterations 70–100, the best, mean, and median solutions exhibit minimal fluctuations, with a consistently narrow standard deviation. This final convergence phase demonstrates that the algorithm has effectively concentrated on high-quality solutions, indicating a stable and refined optimization process. Overall, the ALO’s behavior is well-captured, transitioning from broad exploration with high variance to stable exploitation with lower and more consistent loss values, ultimately converging on optimal or near-optimal solutions with minimized variance.

Table 3 displays the results of the ALO algorithm used to determine the best weights in a TCN by minimizing the loss function. The table contains statistical metrics to evaluate the performance of ALO across various solutions and iterations, aiming to optimize the model. It is a detailed explanation of each column:

**Table 3:** Objective Functions Sample Results regarding Best, Worst, Mean, STV, and Variance.

Iteration	Best	Worst	Mean	Median	STV	Variance
1	6.17855	137.55612	64.95830	55.70171	36.88464	1360.47661
2	0.13748	200.00000	86.20344	88.25383	58.50799	3423.18493
3	0.97504	194.99472	84.85899	74.43265	58.74367	3450.81904
4	2.37588	195.48959	85.37222	83.91306	56.16565	3154.58019
5	4.77585	180.24355	81.53909	86.61172	49.53899	2454.1120
6	0.43533	174.49593	73.20927	79.13677	47.17279	2225.2716
7	0.05961	164.60705	69.37278	67.37450	51.77216	2697.05762
8	8.44240	149.98572	97.56670	76.76302	40.75428	1660.91121
9	1.70184	169.37006	64.26428	59.51051	45.35050	2056.6676
10	0.40235	192.81923	79.37671	70.40963	53.05034	2814.33859
11	0.20153	184.62966	81.53641	83.26765	50.41010	2541.17865
12	7.70500	171.88607	97.38042	107.93917	48.45763	2348.14207
13	0.81597	182.43756	89.18024	82.93629	43.36676	1880.67603
14	1.49385	188.96182	89.56881	97.66188	49.43429	2443.74891
15	0.79754	184.71110	78.79820	77.06317	50.35140	2535.26310
16	1.62096	200.00000	86.82338	97.54626	56.32653	3172.67819
17	1.09277	168.54015	84.62938	98.42873	51.51176	2653.46147
18	0.93705	200.00000	79.01608	72.59351	58.11854	3377.76502
19	1.39603	200.00000	90.45763	82.72940	48.76362	2377.89110
20	1.65143	191.42708	91.00546	96.35240	54.07409	2924.00757

- Best column Represents the minimum loss value achieved by the ALO algorithm for the respective trials or runs. This column highlights the most optimal solutions found by ALO, showing the best fitness (smallest loss) for weights.
- Worst column Indicates the maximum loss value observed during the optimization process. This value reflects the poorest performance of the solutions during the search process.
- The mean column Represents the average loss calculated over all iterations or candidate solutions. It provides an overall measure of the algorithm’s performance stability across runs.
- The median column denotes the middle loss value in the ordered results. It serves as a robust measure of central tendency, minimizing the impact of outliers.
- STV (Standard Deviation) reflects the variation or spread of the loss values. A smaller STV indicates more consistent performance, while a larger STV suggests variability among solutions.
- The variance column measures the spread of loss values around the mean. Like STV, variance highlights the stability of the optimization process. Lower variance indicates a more reliable solution.

There are many important points in Table 3 as follows: The “Best” values in the table (e.g., 6.178855, 0.13748) show that the ALO algorithm successfully minimized the loss function for multiple trials. These values reflect the optimal weights identified for the TCN. A significant difference between the Best and Worst columns (e.g., 0.13748 vs. 200.00000) indicates that while ALO can find excellent solutions, there may be instances where convergence is less effective, emphasizing the importance of fine-tuning ALO parameters. The closeness between the Mean and Median (e.g., Mean = 64.95830, Median = 55.70171) shows the algorithm’s ability to consistently find solutions close to the optimal range. A lower STV value (e.g., 36.88464) implies stability in the solutions, meaning ALO consistently identifies weights with minimal deviation in loss values. The “Variance” column confirms how spread out the results are. Lower variance indicates reliable optimization outcomes.

Fairly consistent quality as it converges, with less influence from poor solutions, particularly in the later stages. The STV starts high at 38.54360 and then fluctuates but decreases as iterations progress. The high STV in early iterations shows high diversity among solutions, indicating broad exploration. STV values gradually decrease over time, showing that the solutions are becoming more similar. A decrease in standard deviation is typical of convergence, as it indicates the algorithm’s focus is narrowing. However, fluctuations in later stages suggest a balance between exploration and exploitation. Variance values follow a pattern like STV, starting at 1485.6088 and decreasing overall, though with some fluctuations. Like STV, variance values start high and decrease, showing convergence as solutions become more consistent. The decreasing trend in variance is a sign of the algorithm homing in on optimal regions, but the fluctuations indicate periodic re-introduction of diversity, likely to prevent premature convergence. It shows the important insights as follows:

- Exploration Phase: In the initial iterations, high variance, STV, and the “Worst” values indicate that the algorithm is broadly exploring the solution space. This is essential in preventing the algorithm from getting stuck in local minima early on.
- Transition Phase: Around middle iterations, there is a notable shift where the “Best” and “Mean” values begin to stabilize, and the variance and STV start decreasing. This indicates that the algorithm is gradually shifting from exploration to exploitation, focusing more on refining the better solutions it has found so far.
- Convergence Phase: in the last iteration, the “Best” solution stabilizes, and both STV and variance remain relatively low, showing that most solutions are now similar in quality. This is a clear sign that the algorithm is converging and refining its search near an optimal solution.
- Intermittent Fluctuations: Small spikes in the “Worst,” “Mean,” and STV values even in later iterations suggest that the algorithm maintains a degree of exploration, which can be beneficial for preventing premature convergence but may slow down convergence slightly if overdone.

**Fig 6:**
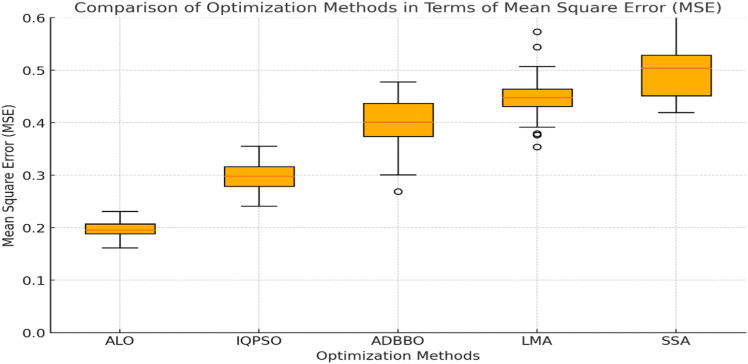
Comparison of optimization methods in terms of MSE.

**Fig 6. illustrates a comparative Mean Square Error (MSE) analysis for five optimization** methods: **ALO**, **IQPSO**, **ADBBO**, **LMA**, and **SSA**. These methods, derived from the related work in our study, are evaluated in terms of their effectiveness in optimizing parameters for deep learning techniques in software defect prediction. The box plot highlights that **ALO** achieves the lowest MSE, indicating its superior performance in minimizing prediction errors, followed by **IQPSO**, which demonstrates competitive results. In contrast, ADBBO, LMA, and SSA exhibit higher MSE values, reflecting relatively less accurate optimization capabilities. The spread of the data and the outliers for each method further emphasize the robustness and stability of ALO in comparison to the others. This comparative analysis underscores the significance of ALO and IQPSO as effective optimization techniques for enhancing predictive accuracy in software defect prediction models.

**Fig 7:**
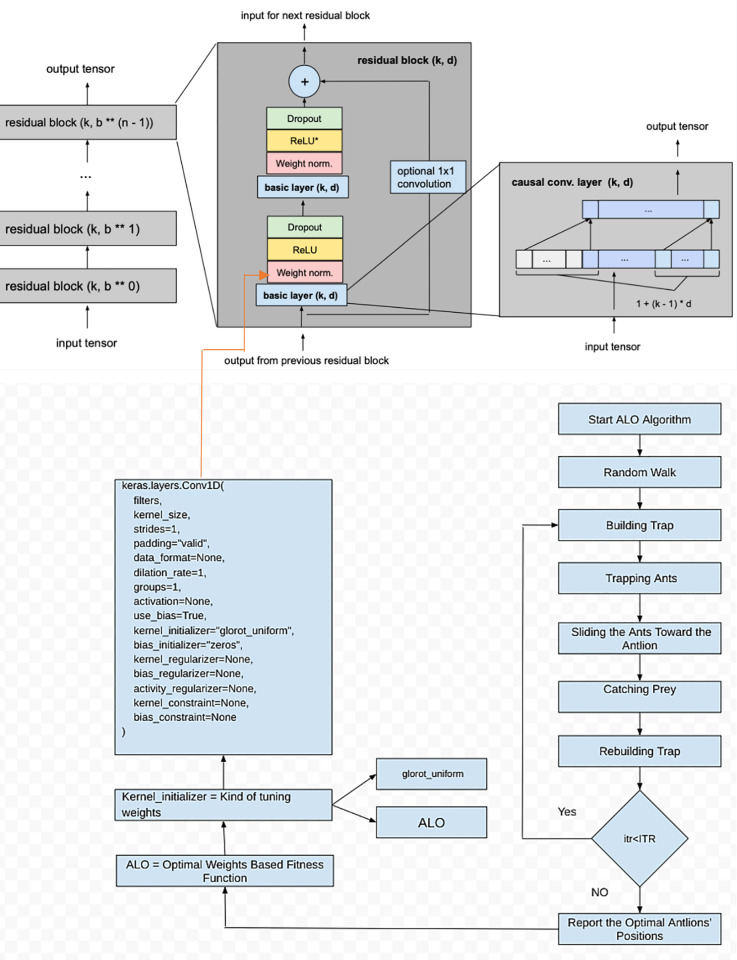
The proposed flow of the ALO-enhanced TCN predictive model.

Fig 7 demonstrates the integration of the ALO algorithm to optimize the weights in a TCN. The process begins with the initialization of the ALO algorithm, where candidate solutions representing the TCN’s weights and biases are generated. The random walk step simulates the exploration of the solution space, allowing the ants (candidate solutions) to move through various weight configurations. In parallel, the antlions (best solutions) create traps that guide the search toward optimal parameters. The ants are progressively “trapped” and adjusted based on their fitness, measured through a loss function derived from the TCN’s performance. The iterative process refines these solutions by continually sliding the ants toward the best-performing antlions, updating the search space with each iteration. If the iteration limit is reached, the ALO algorithm terminates, and the final optimal weights are reported. These optimized weights are then applied to the TCN to improve its performance in processing input data and minimizing prediction errors.

This study utilizes the ALO algorithm to enhance the performance of a TCN model by optimizing its weights. The algorithm initializes the TCN with random weights and sets parameters such as the number of ants (N), antlions (M), and maximum iterations (MaxIter). The optimization process evaluates ants and antlions based on the TCN’s loss function and adjusts their movements using specific equations within a defined search space. Stronger antlions are selected using a roulette wheel method to refine the TCN weights. The fitness of ants and antlions is reassessed after each update, and the process continues until the maximum iterations, or a stopping criterion is met, ensuring optimal parameter tuning for the TCN model.

TCN is used to identify defects in software projects through two distinct approaches. The first approach utilizes a standard TCN model, while the second leverages an optimized TCN-ALO model. A comparative analysis between these models, based on specific criteria derived from the literature [16], is presented in Table 4, highlighting the architectural differences and performance variations. This comparison emphasizes the effectiveness of integrating the ALO algorithm to enhance TCN’s predictive capabilities for software defect detection.

**Table 4:** A structural comparison of the TCN-ALO model and Basic TCN model.

No	Criteria	TCN-ALO	Basic TCN
1	Convolutional Layers	3	3
2	Dilation Rates	Exponential increase: d = [1,2,4]	Exponential increase: d = [1,2,4]
3	Receptive Field	Dynamically adjusted with dilated convolutions	Dynamically adjusted with dilated convolutions
4	Residual Connections	Integrated to improve gradient flow and training stability	Integrated to improve gradient flow and training stability
5	Dropouts	Between residual blocks	Between residual blocks
6	Kernel Size	3	3
7	Training and Optimizer	Adam + Binary cross-entropy	Adam + Binary cross-entropy
8	Activation Function	ReLU + sigmoid (final dense layer)	ReLU + sigmoid (final dense layer)
**9**	**Weights Optimizer**	**ALO**	**Glorot_uniform**
10	Input Shapes	Shape of each Dataset	Shape of each Dataset
11	Output Layer	Fully connected dense layer for classification or regression tasks	Fully connected dense layer for classification or regression tasks

The effectiveness of the suggested TCN model is evaluated through five primary metrics: Accuracy (ACC), Specificity (SPEC), Sensitivity (SENS), and the area under the ROC curve (AUTC), as shown in equations 13, 14, 15, and 16 [43–45].


$$\text{Accuracy }=\frac{TP+TN}{TP+TN+FP+FN}$$ (13)

$$\text{SPEC }=\frac{TN}{TN+FP}$$ (14)

$$\text{SENS }=\frac{TP}{TP+TN}$$ (15)

$$\text{AUTC }=\overset{b}{\underset{a}{\int }}F\left(x\right)dx$$ (16)

**Where:**

- TP = correctly identified data
- TN = correctly rejected data
- FP = incorrectly identified data
- FN = incorrectly identified data
- f(x) = the function that defines the curve.
- a and b = the limits of integration, which are the x-coordinates that bound the area you want to find.
- dx = the integration concerns the variable x.

The outcomes, outlined in Table 5, compares the performance of two methods, T+CN and its improved version, TCN-ALO. TCN-ALO consistently outperforms the baseline TCN in most datasets. Sensitivity, which measures the ability to detect positive cases, shows significant improvement with TCN-ALO, particularly in datasets like Art1 (from 0.4444 to 1.0000) and Ar5 (from 0.5278 to 0.8036). Specificity, Accuracy, and AUTC also exhibit noticeable gains, indicating the enhanced ability of TCN-ALO to correctly predict outcomes and distinguish between classes. Additionally, the Error Rate (ERR) is generally lower for TCN-ALO, further proving its reliability.

**Table 5:** Performance Outcomes of the TCN-ALO against TCN.

Dataset	Method	Sensitivity	Specificity	Accuracy	ERR	AUTC
KC1	TCN	0.1620	0.8837	0.8959	0.1263	0.79
	TCN-ALO	0.2552	0.8944	0.9125	0.1097	0.82
KC2	TCN	0.3656	0.8627	0.8704	0.1419	0.80
	TCN-ALO	0.3959	0.9116	0.9187	0.1035	0.88
KC3	TCN	0.3333	0.8336	0.9090	0.1211	0.71
	TCN-ALO	0.4444	0.8878	0.9670	0.0541	0.95
JM1	TCN	0.1463	0.8833	0.8407	0.1705	0.71
	TCN-ALO	0.0878	0.8977	0.8536	0.1676	0.73
Ar1	TCN	0.4444	0.7935	0.8610	0.1511	0.62
	TCN-ALO	1.0000	0.8911	0.9780	0.0430	0.98
Ar3	TCN	0.3322	0.8397	0.7690	0.2521	0.60
	TCN-ALO	0.4322	1.0000	0.9270	0.1042	0.98
Ar4	TCN	0.1520	0.8726	0.8110	0.2010	0.73
	TCN-ALO	0.6254	0.8972	0.8405	0.1707	0.92
Ar5	TCN	0.5778	0.6633	0.7506	0.2616	0.92
	TCN-ALO	0.5878	0.7863	0.8405	0.1718	0.94
Ar6	TCN	0.1530	0.8694	0.8075	0.2147	0.70
	TCN-ALO	0.3397	0.8792	0.8610	0.1502	0.95
JEdit-4.0	TCN	0.2354	0.7537	0.7398	0.2724	0.67
	TCN-ALO	0.2589	0.8386	0.8159	0.2063	0.88
JEdit-4.2	TCN	0.3486	0.8374	0.8881	0.1119	0.75
	TCN-ALO	0.3986	0.9890	0.8971	0.1023	0.93
JEdit-4.3	TCN	0.5555	0.9698	0.9615	0.0325	0.88
	TCN-ALO	0.6623	0.9973	0.9995	0.0116	0.99
Ant-1.7	TCN	0.4990	0.9229	0.8181	0.2032	0.77
	TCN-ALO	0.5333	0.9876	0.8279	0.1943	0.82
Tomcat-6.0	TCN	0.1838	0.9538	0.9105	0.1052	0.76
	TCN-ALO	0.4322	0.9636	0.9215	0.1207	0.92
CM1	TCN	0.0000	0.9732	0.8790	0.1422	0.77
	TCN-ALO	0.0778	0.9884	0.9133	0.1199	0.88
MC2	TCN	0.5475	0.9322	0.8804	0.1317	0.72
	TCN-ALO	0.6322	0.9585	0.9281	0.1031	0.85
MW1	TCN	0.1322	0.9843	0.9314	0.0797	0.77
	TCN-ALO	0.6324	0.9953	0.9754	0.0467	0.96
PC1	TCN	0.0496	0.9533	0.9471	0.0730	0.75
	TCN-ALO	0.1265	0.9840	0.9592	0.0621	0.82
PC2	TCN	0.7853	0.3245	0.3493	0.0739	0.73
	TCN-ALO	0.8122	1.0000	0.9897	0.0025	0.96
PC3	TCN	0.1322	0.9500	0.9276	0.1167	0.76
	TCN-ALO	0.1433	0.9930	0.9325	0.1029	0.90
PC4	TCN	0.3726	0.9774	0.9397	0.0725	0.90
	TCN-ALO	0.3842	0.9852	0.9419	0.0692	0.93
PC5	TCN	0.1062	0.9849	0.9834	0.0177	0.93
	TCN-ALO	0.1485	0.9947	0.9935	0.0025	0.95

The greatest improvements are observed in datasets such as PC5, where TCN-ALO achieves an Accuracy of 0.9834 and a minimal ERR of 0.0025. Similarly, JEdit-4.3 and Art1 highlight strong gains in Sensitivity and AUTC, reflecting the robustness of the method. While the baseline TCN performs well, the enhancements introduced by TCN-ALO lead to superior overall performance, as reflected by higher AUTC scores across most datasets. In conclusion, TCN-ALO demonstrates significant advancements over TCN, achieving better predictive accuracy, sensitivity, and error reduction across a variety of tested datasets.

Table 6 compares the performance of the TCN-ALO method with and without cross-validation across various datasets, The inclusion of cross-validation generally improves the performance of the model, as evident in most metrics. For example, in KC2, the sensitivity increases from 0.5711 to 0.6495, and the accuracy improves from 0.9187 to 0.9488. Similarly, datasets like Ar3, Ar5, and PC5 show significant gains in Sensitivity, Specificity, and AUTC with cross-validation, indicating better classification performance and generalization.

**Table 6:** Performance Outcomes of the TCN-ALO without and with Cross-Validation.

Dataset	CV	Sensitivity	Specificity	Accuracy	ERR	AUTC
KC1	without	0.3774	0.9456	0.9135	0.1097	0.82
	with	0.4105	0.9813	0.9248	0.1054	0.83
KC2	without	0.5171	0.9341	0.9187	0.1079	0.87
	with	0.6990	0.9820	0.9204	0.0908	0.89
KC3	without	0.3666	0.9163	0.9271	0.0541	0.95
	with	1.0000	0.9531	0.9368	0.0245	0.96
JM1	without	0.0991	0.9009	0.8536	0.1686	0.73
	with	0.0637	0.9895	0.8647	0.1575	0.74
Ar1	without	1.0000	0.9222	0.9682	0.0430	0.97
	with	0.0000	1.0000	0.9716	0.0306	0.99
Ar3	without	0.6222	1.0000	0.9260	0.0965	0.98
	with	0.6322	1.0000	0.9365	0.0943	0.99
Ar4	without	0.7476	0.8351	0.8414	0.1707	0.92
	with	1.0000	0.8598	0.8509	0.1413	0.93
Ar5	without	0.6990	0.9083	0.8425	0.1707	0.94
	with	0.0000	1.0000	0.8804	0.1318	0.95
Ar6	without	0.4509	0.9816	0.8610	0.1502	0.95
	with	0.9083	1.0000	0.9899	0.0202	0.96
JEdit-4.0	without	0.3701	0.9508	0.8159	0.0063	0.88
	with	1.0000	0.9654	0.8256	0.5135	0.90
JEdit-4.2	without	0.1107	0.9713	0.8972	0.1240	0.93
	with	0.7833	1.0000	0.9879	0.0250	0.95
JEdit-4.3	without	0.0000	0.9756	0.9885	0.0227	0.97
	with	0.3666	1.0000	0.9922	0.0121	0.99
Ant-1.7	without	0.2555	0.9796	0.8269	0.1943	0.82
	with	0.4058	0.9963	0.8672	0.1540	0.83
Tomcat-6.0	without	0.6222	0.9358	0.8915	0.1207	0.92
	with	0.6457	0.9858	0.9303	0.0729	0.93
CM1	without	0.1990	0.9496	0.9122	0.1199	0.88
	with	1.0000	0.9617	0.9365	0.0368	0.89
MC2	without	0.8232	0.9707	0.9271	0.0931	0.85
	with	0.8536	1.0000	0.9325	0.0865	0.87
MW1	without	0.8222	0.9413	0.9754	0.0468	0.96
	with	0.9222	0.9963	0.9893	0.0228	0.97
PC1	without	0.1609	0.9663	0.9392	0.0720	0.82
	with	0.1856	0.9987	0.9419	0.0603	0.85
PC2	without	0.2833	1.0000	0.9269	0.0543	0.96
	with	1.0000	0.0009	0.9514	0.042	0.97
PC3	without	0.2259	0.9564	0.9103	0.1029	0.90
	with	0.5733	0.9665	0.9236	0.0978	0.92
PC4	without	0.4564	0.9774	0.9310	0.0702	0.93
	with	0.4778	0.9994	0.9514	0.0608	0.94
PC5	without	0.2308	0.9569	0.9523	0.0286	0.95
	with	1.0000	0.9758	0.9687	0.0125	0.97

Moreover, in certain datasets like Ar6 and MW1, the use of cross-validation leads to a perfect sensitivity score of 1.0000, highlighting the robustness of the model in detecting all positive cases. A notable reduction in Error Rate (ERR) is also observed across most datasets when using cross-validation. For instance, in PC5, the ERR drops from 0.0208 to 0.0125. These improvements demonstrate that cross-validation helps the model generalize better across datasets, leading to more reliable and consistent performance. Overall, the table highlights the value of cross-validation in enhancing TCN-ALO’s performance across diverse datasets.

### Outcomes of the TCN-ALO against state-of-the-art

Table 7 summarizes state-of-the-art methods for identifying defects in software projects, highlighting a variety of algorithms and their respective references. The methods are grouped by their abbreviations and cover diverse machine learning, deep learning, and statistical techniques.

**Table 7:** State-of-the-art method for revealing defects in software projects.

Abbreviation	Method Name	Reference	Abbreviation	Method Name	Reference
KNN-GA	K Nearest Neighbour-Genetic Algorithm	[46]	ANN-PCA	ANN-Principal Component Analysis	[48]
SVM	Support Vector Machine	[29]	PCA-SVM	PCA-Support vector Machine	[49]
L-SVM	Lagrangian Support Vector Machine	[32,47]	CNN	convolutional neural network	[50]
LS-SVM	Least Squares Support Vector Machine	[32,47]	GRU	Gated recurrent unit	[50]
NB	Naïve Bayes	[32,47]	BI-LSTM	Bidirectional long short-term memory	[50]
LDA	Linear Discriminant Analysis	[32,47]	DF	Deep forest	[51]

Table 8 compares the performance of TCN-ALO against seven other techniques (KNN-GA, SVM, L-SVM, LS-SVM, NB, LDA, and ANN-PCA) across four datasets (KC1, JM1, PC3, and PC4) using the AUTC metric. TCN-ALO consistently outperforms all other techniques across all datasets, achieving the highest scores (e.g., 0.82 for KC1, 0.73 for JM1, 0.90 for PC3, and 0.93 for PC4). While other techniques such as LS-SVM and ANN-PCA also perform well, particularly on datasets like PC3 and PC4, the results highlight the robustness and effectiveness of TCN-ALO in delivering superior performance in varied scenarios. The performance differences among methods indicate dataset-specific variations but confirm TCN-ALO’s consistent advantage.

**Table 8:** Outcomes of the TCN-ALO against seven techniques to AUTC.

Dataset	KNN-GA	SVM	L-SVM	LS-SVM	NB	LDA	ANN-PCA	TCN-ALO
KC1	0.71	0.77	0.77	0.78	0.77	0.79	0.79	0.82
JM1	0.70	0.69	0.70	0.71	0.70	0.71	0.71	0.73
PC3	0.78	0.78	0.85	0.84	0.82	0.83	0.87	0.90
PC4	0.88	0.90	0.89	0.87	0.86	0.89	0.90	0.93

Table 9 compares the performance of two methods, ANN-PCA and TCN-ALO, across four datasets (KC1, JM1, PC3, and PC4) based on three metrics: sensitivity, specificity, and accuracy. Sensitivity, which measures the model’s ability to correctly identify positive cases, shows that TCN-ALO outperforms ANN-PCA in most datasets, such as KC1 (0.2330 vs. 0.1161) and PC3 (0.1000 vs. 0.8848). However, ANN-PCA performs better on sensitivity in the JM1 and PC4 datasets. Specificity, which evaluates the ability to correctly identify negative cases, consistently favors TCN-ALO, with a substantial improvement in datasets like PC3 (0.9718 vs. 0.3111) and PC4 (0.9630 vs. 0.5350). For accuracy, TCN-ALO shows superior performance across all datasets, particularly excelling in KC1 (0.9125 vs. 0.8702) and PC4 (0.9419 vs. 0.9087). Overall, TCN-ALO demonstrates more balanced and higher performance across the datasets, making it the more robust method compared to ANN-PCA.

**Table 9:** Outcomes of the TCN-ALO against ANN-PCA.

Dataset	Method	Sensitivity	Specificity	Accuracy
KC1	ANN-PCA	0.1161	0.8733	0.8702
	TCN-ALO	0.2552	0.8944	0.9125
JM1	ANN-PCA	0.8704	0.1726	0.8201
	TCN-ALO	0.0878	0.8977	0.8536
PC3	ANN-PCA	0.8848	0.3111	0.8831
	TCN-ALO	0.1433	0.9930	0.9325
PC4	ANN-PCA	0.8676	0.5350	0.9087
	TCN-ALO	0.3842	0.9852	0.9419

Table 10 presents a comparative analysis of the outcomes achieved by the TCN-ALO model versus the PCA-SVM method across various datasets. Sensitivity measures the model’s ability to correctly identify defective software modules, while specificity evaluates its capability to identify non-defective ones. Accuracy represents the overall classification performance, and AUTC provides a summary metric combining sensitivity and specificity. TCN-ALO consistently outperforms PCA-SVM across most datasets, achieving higher accuracy and AUTC values. For instance, in the Jedit-4.3 dataset, TCN-ALO achieves an accuracy of 99.95% and an AUTC of 0.99, significantly surpassing PCA-SVM. Similarly, TCN-ALO demonstrates superior sensitivity and specificity in datasets like Ar1 and Ant-1.7. These results highlight the robustness and effectiveness of TCN-ALO in defect prediction tasks, showcasing its ability to deliver higher predictive accuracy and better handling of imbalanced datasets compared to PCA-SVM. The table underscores TCN-ALO’s capability to provide more reliable defect classification for diverse software projects.

**Table 10:** Outcomes of the TCN-ALO against PCA-SVM.

Dataset	Method	Sensitivity	Specificity	Accuracy	AUTC
Ar1	PCA-SVM	0.445	0.8800	0.8480	0.66
	TCN-ALO	1.0000	0.8911	0.9780	0.98
Ar3	PCA-SVM	1.0000	0.7000	0.7142	0.81
	TCN-ALO	0.4322	1.0000	0.9270	0.98
Ar4	PCA-SVM	0.5400	0.6868	0.7857	0.89
	TCN-ALO	0.6254	0.8972	0.8405	0.92
Ar5	PCA-SVM	0.4187	0.6998	0.8194	0.91
	TCN-ALO	0.5878	0.7863	0.8405	0.94
Ar6	PCA-SVM	0.3067	0.8298	0.8094	0.82
	TCN-ALO	0.3397	0.8792	0.8610	0.95
Jedit-4.0	PCA-SVM	0.1918	0.7971	0.7169	0.84
	TCN-ALO	0.2589	0.8386	0.8159	0.88
Jedit-4.2	PCA-SVM	0.1009	0.9067	0.8248	0.90
	TCN-ALO	0.3986	0.9890	0.8971	0.93
Jedit-4.3	PCA-SVM	0.5252	0.9540	0.9462	0.81
	TCN-ALO	0.6623	0.9973	0.9995	0.99
Ant-1.7	PCA-SVM	0.1011	0.9343	0.7846	0.77
	TCN-ALO	0.5333	0.9876	0.8279	0.82
Tomcat 6.0	PCA-SVM	0.3054	0.8803	0.8382	0.86
	TCN-ALO	0.4322	0.9636	0.9215	0.92

Table 11 compares the accuracy of the TCN-ALO model against three alternative techniques—CNN, GRU, and Bi-LSTM—across multiple datasets. Accuracy values indicate the proportion of correctly classified instances. Across all datasets, TCN-ALO consistently achieves the highest accuracy, demonstrating its superiority over the other methods. For example, in the KC3 dataset, TCN-ALO achieves an accuracy of 96.70%, significantly higher than CNN (77.83%), GRU (77.55%), and Bi-LSTM (79.03%). Similarly, in the MW1 dataset, TCN-ALO reaches an accuracy of 97.54%, outperforming CNN (81.14%), GRU (70.83%), and Bi-LSTM (82.46%). These results indicate the robustness and efficiency of TCN-ALO in handling diverse datasets and providing accurate defect predictions. The consistent superiority of TCN-ALO highlights its effectiveness in leveraging temporal convolution and Antlion Optimization for software defect prediction tasks, surpassing both convolutional and recurrent-based approaches. This table emphasizes TCN-ALO’s ability to outperform state-of-the-art techniques in terms of predictive accuracy.

**Table 11:** Outcomes of the TCN-ALO against three techniques to accuracy.

Dataset	CNN	GRU	BI-LSTM	TCN-ALO
CM1	0.8142	0.8112	0.8113	0.9133
JM1	0.7724	0.7712	0.7918	0.8536
KC2	0.8265	0.8240	0.8463	0.9187
KC3	0.7783	0.7753	0.7903	0.9670
MC2	0.7051	0.7063	0.7203	0.9281
MW1	0.8114	0.7083	0.8246	0.9754
PC1	0.8765	0.8740	0.8919	0.9592
PC2	0.9083	0.9089	0.9183	0.9897
PC3	0.4128	0.4364	0.3676	0.9325
PC4	0.8637	0.8654	0.8815	0.9419
PC5	0.8526	0.84578	0.8902	0.9935

Table 12 compares the AUTC outcomes of the TCN-ALO model against the DF method across multiple datasets, with and without cross-validation. The results consistently demonstrate that TCN-ALO achieves higher AUTC values than DF in all scenarios, both with and without cross-validation. For instance, in the Ant-1.7 dataset without cross-validation, TCN-ALO achieves an AUTC of 0.82, compared to DF’s 0.70; with cross-validation, TCN-ALO reaches 0.83, while DF scores 0.77. Similar trends are observed in the Jedit datasets. For Jedit-4.3, TCN-ALO achieves an outstanding AUTC of 0.97 without cross-validation and 0.99 with cross-validation, outperforming DF’s 0.90 and 0.91, respectively. These results highlight the robust and consistent performance of TCN-ALO in improving defect prediction accuracy, regardless of the evaluation scenario. The table underscores TCN-ALO’s superior ability to enhance AUTC through its combination of Temporal Convolutional Network and Antlion Optimization, making it more effective than traditional DF techniques in defect prediction tasks.

**Table 12:** Outcomes of the TCN-ALO against DF to AUTC.

Dataset	Cross-validation	Method	AUTC	Cross-validation	Method	AUTC
Ant-1.7	Without	DF	0.70	With	DF	0.77
		TCN-ALO	0.82		TCN-ALO	0.83
Jedit-4.0	Without	DF	0.83	With	DF	0.84
		TCN-ALO	0.88		TCN-ALO	0.90
		TCN-ALO	0.97		TCN-ALO	0.99
Jedit-4.2	Without	DF	0.86	With	DF	0.88
		TCN-ALO	0.93		TCN-ALO	0.95
Jedit-4.3	Without	DF	0.90	With	DF	0.91

### Statistical analysis of the TCN against TCN-ALO

In this article, we will conduct a comprehensive analysis using the T-test to compare various algorithms. This comparison will focus on evaluating their performance through regression and correlation metrics. A key aspect of our analysis is the P-value, which indicates the probability that our hypothesis is correct based solely on random occurrences.

Table 13 presents a comprehensive statistical analysis comparing the performance of TCN and TCN-ALO across various datasets. Key metrics include average accuracy, standard deviation (std), mean standard error, and p-value to assess statistical significance, as follows:

**Table 13:** The statistical analysis of TCN and TCN-ALO.

Dataset	Method	Average	Standard Deviation (std)	Mean std Error	P-Value	Dataset	Method	Average	Standard Deviation (std)	Mean std Error	P-Value
KC1	TCN	0.8934	0.0082	0.0013	0.00	Ant-1.7	TCN	0.7935	0.0052	0.0013	0.00
							TCN-ALO	0.8269	0.0038	0.0009	
	TCN-ALO	0.9212	0.0042	0.0011							
KC2	TCN	0.8623	0.0053	0.0009	0.00	Ar1	TCN	0.8347	0.1325	0.0019	0.00
							TCN-ALO	0.9925	0.0001	0.0001	
	TCN-ALO	0.9176	0.0016	0.0003							
KC3	TCN	0.8310	0.0021	0.0032	0.00	Ar3	TCN	0.9501	0.0105	0.0042	0.00
							TCN-ALO	0.9816	0.0001	0.0002	
	TCN-ALO	0.9431	0.0012	0.0017							
MC2	TCN	0.7245	0.0195	0.0045	0.00	Ar4	TCN	0.9880	0.0039	0.0005	0.00
							TCN-ALO	0.9995	0.0001	0.0001	
	TCN-ALO	0.8621	0.0038	0.0008							
MW1	TCN	0.8568	0.0113	0.0068	0.00	Ar5	TCN	0.9357	0.0331	0.0011	0.00
							TCN-ALO	0.9519	0.0001	0.0001	
	TCN-ALO	0.9718	0.0023	0.0007							
PC1	TCN	0.8080	0.0059	0.0038	0.00	Ar6	TCN	0.7475	0.1205	0.0029	0.00
							TCN-ALO	0.9983	0.0001	0.0001	
	TCN-ALO	0.9142	0.0020	0.0016							
PC2	TCN	0.9078	0.0449	0.0028	0.00	CM1	TCN	0.8351	0.0199	0.0001	0.00
							TCN-ALO	0.9545	0.0019	0.0001	
	TCN-ALO	0.9989	0.0009	0.0001							
PC3	TCN	0.8420	0.0024	0.0014	0.00	Jedit-4.0	TCN	0.7766	0.0033	0.0003	0.00
							TCN-ALO	0.8752	0.0024	0.0001	
	TCN-ALO	0.8937	0.0028	0.0006							
PC4	TCN	0.9085	0.0021	0.0008	0.00	Jedit-4.2	TCN	0.9839	0.0240	0.0013	0.00
							TCN-ALO	0.9928	0.0001	0.0002	
	TCN-ALO	0.9269	0.0011	0.0002							
PC5	TCN	0.9806	0.0033	0.0009	0.00	Jedit-4.3	TCN	0.9535	0.0401	0.0019	0.00
							TCN-ALO	0.9964	0.0006	0.0001	
	TCN-ALO	0.9926	0.0004	0.0004							
JM1	TCN	0.8015	0.0069	0.0014	0.00	Tomcat- 6.0	TCN	0.8956	0.0292	0.0041	0.00
							TCN-ALO	0.9689	0.0021	0.0001	
	TCN-ALO	0.8301	0.0056	0.0012

- Average Accuracy: TCN-ALO consistently achieves higher average accuracy compared to TCN across all datasets. For example, in the KC3 dataset, TCN achieves an average accuracy of 0.8310, while TCN-ALO outperforms it with an accuracy of 0.9431. Similarly, for the PC2 dataset, TCN-ALO achieves an impressive 0.9989 compared to TCN’s 0.9078.
- Standard Deviation (Std): TCN-ALO generally has a lower standard deviation compared to TCN, indicating more stable and consistent performance. For instance, in the MW1 dataset, TCN-ALO has a standard deviation of 0.0023, whereas TCN’s standard deviation is 0.0113.
- Mean Standard Error: TCN-ALO exhibits smaller mean standard errors, reflecting higher reliability in its accuracy results. For example, in the Ar4 dataset, TCN-ALO shows a mean standard error of 0.0005, significantly lower than TCN’s 0.0039.
- P-Value: The p-values for all datasets are 0.00, indicating that the improvements made by TCN-ALO over TCN are statistically significant. This demonstrates that the performance difference is not due to random chance.
- Diverse Datasets: Across datasets like KC1, Ant-1.7, PC1, and Tomcat 6.0, TCN-ALO shows superior performance, making it highly robust and generalizable across diverse scenarios.

## Discussion

This study demonstrates the significant advantages of combining TCN with ALO for software defect prediction. By addressing the challenges posed by high-dimensional datasets and imbalanced classes, the TCN-ALO model delivers superior performance compared to traditional methods such as Random Forests, CNNs, GRUs, and Bi-LSTMs. The results indicate consistent improvements across critical metrics, including accuracy, sensitivity, specificity, and AUCT, highlighting the robustness of this hybrid approach. These improvements underscore the potential of integrating metaheuristic optimization techniques with temporal deep learning architectures to tackle complex software defect prediction problems effectively.

One of the key contributions of this study is the successful integration of ALO to optimize the parameters of the TCN model. Unlike traditional optimization techniques that often suffer from issues like local minima entrapment and slow convergence, ALO effectively balances exploration and exploitation during the optimization process. This balance enables the model to achieve a globally optimal set of parameters, leading to enhanced predictive accuracy and stability across diverse datasets. The reduced standard deviation and lower error rates observed in this study further reinforce ALO’s capability to fine-tune deep learning models efficiently.

Another strength of the TCN-ALO model is its scalability and adaptability to various datasets. Extensive evaluation across datasets such as NASA, PROMISE, and open-source projects demonstrates the model’s ability to generalize beyond a single domain. The cross-validation results further validate its effectiveness in mitigating overfitting and ensuring consistent performance. These findings highlight the practicality of the TCN-ALO model for real-world applications, where data diversity and generalization are critical challenges. By improving sensitivity and specificity, the model proves particularly effective in identifying defective modules while minimizing false negatives and false positives.

The study also emphasizes the importance of computational efficiency in defect prediction tasks. TCN-ALO achieves high performance with fewer retraining cycles, reducing training time and computational resources required for large-scale software projects. This efficiency makes it an attractive option for deployment in industrial settings, where resource constraints and time-to-market considerations play a crucial role. Furthermore, the model’s ability to handle imbalanced datasets with higher sensitivity and AUCT metrics ensures its utility in practical scenarios, where defect-prone modules are often underrepresented.

## Conclusions

This study proposed a hybrid model that combines TCN with ALO to enhance the accuracy and efficiency of software defect prediction. By utilizing TCN’s capability to automatically extract relevant features and incorporating ALO for optimized parameter tuning, the model achieved significant improvements in accuracy, sensitivity, and specificity across diverse datasets. Comparative evaluations confirmed the model’s superiority over standard TCNs and state-of-the-art techniques such as Recurrent Neural Networks and Deep Forest, with accuracy improvements of up to 26.7%. Despite these promising results, certain limitations need to be addressed. One challenge lies in the higher computational cost associated with ALO compared to gradient-based optimization methods, which may reduce its practicality for real-time applications in large-scale software systems. Additionally, the model was primarily validated on publicly available datasets, which may not fully capture the diversity and complexity of real-world software projects. Future research should focus on extending the application of this model to a broader range of datasets, including those specific to industry domains, to evaluate its generalizability and effectiveness in diverse scenarios. Moreover, optimizing the ALO algorithm to reduce computational overhead while maintaining or enhancing its performance could improve the model’s scalability. Exploring the integration of alternative metaheuristic algorithms or hybrid optimization approaches could further augment the model’s predictive capabilities. Lastly, real-world evaluations, such as case studies or pilot implementations in software development environments, are necessary to assess the model’s practical applicability and potential impact on improving software quality assurance practices. These directions would pave the way for refining the model and making it a more versatile tool in software defect prediction.
